# Implementation of a Continuous Video Monitoring Program to Decrease Inpatient Falls in a Long-Term Acute Care Hospital Setting: A Prospective Observational Cohort Study

**DOI:** 10.1097/AJN.0000000000000197

**Published:** 2025-11-20

**Authors:** Lisa Kalafus, Henry Charles Hrdlicka, Jennifer Lombardi, Samantha Proctor, June Napolitano, Nicole Morrill

**Affiliations:** **Lisa Kalafus** is the chief nursing officer at Gaylord Specialty Healthcare in Wallingford, CT, where **Henry Charles Hrdlicka** is the director of research, **Jennifer Lombardi** is the telesitting coordinator and a nurse supervisor, and **June Napolitano** and **Nicole Morrill** are nurse managers. **Samantha Proctor** is a graduate medical student at the Campbell University Jerry M. Wallace School of Osteopathic Medicine in Buies Creek, NC, as well as a research apprentice at Gaylord Specialty Healthcare. Leasing costs for the continuous video monitoring devices and system software used in the study were paid by the study site; the manufacturer did not sponsor the research. The authors wish to acknowledge the telesitter technicians as well as the team members from the information technology, quality and safety, and nursing departments who provided input on implementation of the program and helped to maintain the equipment. Contact co–first authors: Lisa Kalafus, lkalafus@gaylord.org, and Henry Charles Hrdlicka, hhrdlicka@gaylord.org. The authors have disclosed no potential conflicts of interest, financial or otherwise.

**Keywords:** cost reduction, fall prevention, inpatient falls, long-term acute care hospital, patient safety, video monitoring, virtual monitoring

## Abstract

**Background:**

Continuous video monitoring programs have been found to reduce inpatient falls and 1:1 sitter use in the short-term acute care hospital setting. But the impact and potential benefits of such programs in the long-term acute care hospital (LTACH) setting are still unknown.

**Purpose:**

The goal of this study was to track the implementation of a continuous video monitoring program in an LTACH setting and evaluate its impact on inpatient falls and 1:1 sitter use, as well as on associated costs.

**Methods:**

A prospective observational cohort study design was used. Prospective data were collected from patients who were admitted to an LTACH in the northeastern United States and subsequently enrolled in a continuous video monitoring program during the 20-month period of February 1, 2021, through September 30, 2022. Primary outcome measures, including inpatient falls and 1:1 sitter hours, were then compared to 20 months of historical data, from June 1, 2019, through January 31, 2021, which were collected through chart review.

**Results:**

Following development and implementation of the continuous video monitoring program, the mean rate of inpatient falls decreased significantly, from 17.2 falls per month in the historical reference period to 12.9 falls per month during the study period *(P =* 0.02). Similarly, the mean number of 1:1 sitter hours decreased from 1,428 hours per month during the reference period to 140 hours per month during the study period (*P* < 0.001); when converted to full-time equivalents (FTEs), this translated to a decrease from 8.2 1:1 sitter FTEs during the reference period to 0.8 1:1 sitter FTEs in the study period. Cost analysis indicated that the reduced labor costs and fall rate during the study period led to estimated total cost savings of over $3.2 million.

**Conclusions:**

Both patients and the hospital benefited from the implementation of the continuous video monitoring program. Continuous video monitoring was found to be a cost-effective way to reduce inpatient falls, decrease 1:1 sitter use, and improve patient safety in the LTACH setting.

Patient falls are a major safety concern and a leading cause of preventable injury in all inpatient hospital settings. An estimated 700,000 to 1 million hospitalized patients experience a fall annually, with 30% to 50% of these falls resulting in significant injury and about 1% resulting in death.^1^-^3^ Compared to other inpatient settings, long-term acute care hospitals (LTACHs) face particular challenges in this regard, considering their patients have longer planned lengths of stay averaging 25 days or more and require complex medical care and intensive rehabilitation.^4^,^5^ Because early mobilization is a common goal from the time of LTACH admission, these patients also tend to have a robust mobility plan of care. But they are often at greater risk for falls as they navigate unexpected changes in their physiologic state and work to regain function.

To address this increased risk, LTACHs implement many fall risk–reducing strategies, including post-fall huddles and debriefings; “intentional” rounding; moving patients to a designated observation room; bed, chair, and seat belt alarms; and self-release belts.^6^,^7^ For patients who demonstrate high impulsivity, react poorly to other strategies, and are at high risk for falling, the use of a 1:1 sitter may be necessary.^8^

Assigned 1:1 sitters provide support and can quickly intervene to prevent or redirect dangerous behaviors that could result in a fall. While 1:1 sitter programs have been shown to effectively prevent falls, they are resource-intensive and quite costly.^9^,^10^ Often, 1:1 sitters are made available by reassigning support staff from the patient care team, which can have an overall negative impact on patient safety.^11^ In other instances, staff may be asked to work additional shifts or overtime to accommodate the need for sitters. Meeting this need exacerbates existing workforce shortages. Moreover, one recent estimate puts the cost of 24-hour 1:1 sitter use at about $561 per patient day, which is detrimental to hospital finances.^9^ It's not surprising that hospital systems have begun looking for alternative solutions,^12^ such as patient surveillance modalities.

One such modality is closed circuit television monitoring, a one-way video monitoring option. Closed circuit television cameras are installed in select patient rooms, and a monitoring station is typically set up at a nursing station to give frontline staff a direct, live view of these patients. Although the video feed is continuous, it isn't necessarily monitored continuously. If a behavior of concern is observed, staff must then leave the monitoring station to attend to the patient, leaving the monitoring station unattended.

In contrast, continuous video monitoring systems (also called continuous virtual monitoring systems) offer one-way video and two-way audio communication capabilities. These systems make use of in-room fixed or mobile telemonitor devices that can be relocated and reassigned based on patient needs. They allow a centralized team of sitters to continuously monitor their patients around the clock, interact with the patients, speak with them directly via two-way audio to address and redirect behaviors of concern, and immediately alert floor staff to behaviors of concern or anticipated concern.^13^,^14^ While such systems have been found to reduce inpatient falls and 1:1 sitter use in the short-term acute care hospital setting, the impact of their use in the LTACH setting is unknown.

To address inpatient fall concerns, address staffing challenges, and reduce 1:1 sitter costs, a continuous video monitoring program was implemented at the study site. The program included mobile telemonitor devices that were set up in select patient rooms and provided real-time continuous observation by a single telemonitor technician per shift. The team of technicians then monitored and interacted with multiple high-risk patients 24/7. It was hypothesized that this program would positively impact inpatient fall rates and reduce the resource strain and financial burden of 1:1 sitters in the LTACH environment.

**Study purpose**. The goal of this study was to track the implementation of a new continuous video monitoring program in an LTACH setting and evaluate its impact on the primary outcomes of inpatient falls and 1:1 sitter use, as well as on associated costs.

## METHODS

**Study design and setting**. The study used a prospective observational cohort study design. Prospective data were collected over 20 months (February 1, 2021, through September 30, 2022). Historical data for primary outcomes were retrospectively collected for the 20 months immediately preceding the study period (June 1, 2019, through January 31, 2021) and were used as comparators. All study activities were conducted at Gaylord Specialty Healthcare, a 137-bed LTACH located in Wallingford, Connecticut. This facility comprises six nursing units that include two step-down progressive care units, two telemetry-enabled medically complex care units, and two rehabilitation units. Prior to data collection, the study was reviewed and granted exempt status by the study site's institutional review board.

**Sample**. Patient eligibility and device assignment for the continuous video monitoring program were based on criteria similar to those for 1:1 sitter use, including reduced alertness, heightened impulsivity, limited awareness of limitations, impaired bladder or bowel management, cognitive impairment, and high fall-risk score. A formal clinical algorithm was developed around these factors to determine patient eligibility, in order to properly assign devices to the patients with the highest need.

**Equipment**. The continuous video monitoring devices were introduced in two waves. Ten devices were implemented on January 26, 2021; two more were added in October. All 12 were AvaSure Guardian mobile devices, which were leased from the manufacturer along with the system software. Each device was equipped with one-way video and two-way audio communication capabilities.

**Program adoption and implementation**. Various steps were taken to aid the program's success. First, staff initially hired for the technician role underwent training provided by the device manufacturer. At the end of their training, trainees took a competency test. Upon successful completion, as designated “super-users,” they could then disseminate their training to other staff. A total of three full-time telemonitor technicians were on duty on any given day at one per shift; a fourth technician was also available to cover open shifts. Nurse educators, nurse supervisors, and nurse managers also completed the training and passed the competency test in order to support the program's implementation and assist in troubleshooting.

Second, before the program launched, standardized criteria and workflows for various elements were created. These included device initiation and reassignment criteria; patient discontinuation criteria; device checkout, return, and wait-list processes; telemonitor technician intervention guidelines that took into account the specific patient safety concerns of the LTACH study site; and telemonitoring documentation processes and guidelines.

*Device initiation, device reassignment, and patient discontinuation criteria*. Devices were initially stored in a centralized location next to the telemonitor technicians' office. Once all 12 devices were available, and whenever the hospital was at full 137-bed capacity, there was approximately one device per 11 patients. As such, protocols for determining a patient's eligibility for the program and initial device assignment, and for subsequent decisions to discontinue a patient from the program and reassign a device, were of utmost importance. As noted above, a formal algorithm was developed to determine patient eligibility and make initial device assignments. Similarly, a triage algorithm was created to assist with patient discontinuation and device reassignment to patients with greater needs. Using these algorithms, along with input from the telemonitor technician on duty, the house nurse supervisor made the final decisions regarding device assignment and reassignment, and patient discontinuation.

*Device checkout, return, and wait-list processes*. To request a device, direct care nurses completed a “ticket to monitor,” outlining the patient's needs and the reason for continuous video monitoring. The completed ticket was then given to the house nurse supervisor on shift, who reviewed the request using the established algorithms. If a device was available, the house nurse supervisor retrieved the device, logged the equipment ID on the ticket to monitor, informed the technician of the incoming program enrollment, and brought the device to the patient's room. If a device was not readily available, the house nurse supervisor reviewed the devices in use and the assigned patients. If it was determined that the new requesting patient had a greater need than a patient currently being monitored, the house nurse supervisor reassigned the device to the patient with the greater need. If the new requesting patient's needs were determined not to be greater than those of any patient currently being monitored, the new requesting patient was placed on a wait list until a device became available. Both the list of currently assigned devices and the wait list were reviewed at least once per shift, or more frequently as needed.

*Telemonitoring documentation processes and guidelines*. Upon device assignment, the telemonitor technician on duty first input the necessary information from the ticket to monitor into the device and the monitoring program database. This information included patient room number, patient age, monitoring start time, clinician notes, reason for monitoring, and potential adverse events of concern. To protect patient identity, no patient names were recorded in the program database; instead, each patient was assigned an automatically generated, random identifier.

Once patients were assigned a device, the system software then logged all interactions between the telemonitor technicians and these patients. The logs reflected the technicians' inputs and how they categorized each interaction. The main category of interest was labeled “adverse event avoided.” Anytime a technician redirected or otherwise prevented an unsafe behavior that could have led to a fall or other adverse event, it was recorded as a potential adverse event avoided. When a patient discontinuation occurred, the technician logged the reason and the date. In addition to the digital records retained in the program database, the technicians also kept paper records. These were used to log each patient's status and to further document any incidents of concern, and served as backup in the event that the digital records became lost or corrupted.

Each patient––or, if the patient was cognitively impaired, their family members––was given advance notice of device implementation, with an explanation of why this was recommended and with the option to choose another appropriate risk-mitigation strategy. Patients and their families also had the option to refuse continuous video monitoring after the device was put in place.

**Data tracking and analysis**. *Inpatient falls and 1:1 sitter use*. Inpatient falls were evaluated using the institutional monthly fall reports, which detailed the events leading up to and following the fall. Fall reports collected during both the historical reference and the study periods were evaluated. Inpatient falls were reported as total falls per month and falls per 1,000 patient-days. The total number of 1:1 sitter hours was logged by the house nurse supervisor at the end of each shift. At the end of each month, these were totaled and reported to the research team and other key stakeholders (including the chief nursing officer and nursing supervisors) for review. At this point, 1:1 sitter hours were reported as total hours, hours per 1,000 patient-days, and full-time equivalents (FTEs), based on the standard 2,080 hours per year.

*Chart review, device reports, and patient satisfaction*. The institutional monthly fall reports were supplemented with targeted chart review to collect patients' demographic information (age and sex), diagnosis, and continuous video monitoring status at the time of the fall (if applicable). In addition, monthly device reports were collected. These included system on–time (program enrollment and device assignment) and system off–time (device discontinuation); time and type of technician–patient interactions; adverse events avoided; reason for monitoring, including potential adverse events of concern (such as falls, elopement, and medical device maintenance); and reason for patient discontinuation or reassignment (or both). For the purpose of analysis, and as appropriate, events were described in terms of total events, mean frequency of events per month, mean frequency of events per 1,000 patient-days (days in hospital), or mean frequency of events per 1,000 monitored patient-days (days on continuous video monitoring).

At the study site, after discharge, all patients were sent a patient satisfaction survey by a third-party vendor. During the study period, the following two questions were added to the survey:

“Question 1: Did you have a continuous video monitoring device during your stay?”

“Question 2: If you had a continuous video monitoring device, was your experience positive?”

If a respondent answered question 1, their responses to both questions were shared with the research team; if they did not answer question 1, then no responses were shared with the research team.

*Statistical analysis* was conducted using GraphPad Prism, version 10.2.0. For total number of events (inpatient falls and 1:1 sitter hours), a two-tailed *z* test was performed. For data considered by month or in terms of 1,000 patient-days or FTEs, an independent samples *t* test was performed to compare the reference and study period groups. For comparing the proportion of falls with injury to those without injury, a chi-square (χ^2^) test was used. Significance was set at *P* ≤ 0.05 for all statistical tests.

*Cost analysis*. Four cost categories were considered: total 1:1 sitter hours and the estimated associated cost, the cost of staffing the telemonitor technician position at 4.2 FTEs, the cost of leasing the program equipment, and the estimated costs associated with inpatient falls. For 1:1 sitter hours, a weighted average hourly rate of $25 per hour was used, which took into account shift differentials and overtime pay. For the telemonitor technicians' salary estimates, an average hourly rate of $21 per hour was used. Equipment leasing costs were based on an average yearly expense of $89,000 per year for two years, or $178,000 total for the study period. (Although the study was 20 months, the service contract required payment for two full years.) The average direct cost associated with each inpatient fall was set at $35,365 per fall, in accordance with a multisite study conducted by Dykes and colleagues.^15^ Expenses associated with the information technology (IT) department's support and Wi-Fi infrastructure improvements were not included in the cost analysis, as those expenses were accrued either as part of individual salaried roles or as part of larger hospital initiatives.

## RESULTS

**Sample characteristics**. A total of 448 patients were enrolled in the continuous video monitoring program during the study period. Of these, 287 (64.1%) were male, and 161 (35.9%) were female. The mean age was 64.6 years (range, 16-90 years), and the mean duration of continuous video monitoring was 13.1 days. Patient conditions of concern at the time of program enrollment were categorized as one of the following: altered mental status (AMS), including delirium and dementia (14.1%); AMS and COVID-19 droplet precautions (1.3%); AMS and other conditions (0.4%); brain injury or stroke (54.5%); brain injury or stroke and AMS (11.4%); brain injury or stroke, AMS, and COVID-19 droplet precautions (0.2%); brain injury or stroke and COVID-19 droplet precautions (1.3%); COVID-19 droplet precautions (3.6%); or other conditions (13.2%), which included being at risk for respiratory failure, seizure, or medical device interference or dislodgment, among others. (Although patients on COVID-19 or other droplet precautions aren't typically assigned a 1:1 sitter, some who were high acuity albeit at lower fall risk were enrolled in the program. This allowed technicians to observe for and redirect high-risk behaviors, so that floor staff either didn't have to don personal protective equipment to enter the room, or at least were alerted sooner to such behaviors.) Primary reasons for patient discontinuation from the program included improved behavior (50.7%), LTACH discharge (23.2%), device reassignment to a higher-need patient (10%), and emergent transfer to an acute care hospital (4.7%). For more details, see Table 1.

**Table 1.** Sample Characteristics (N = 448)

Characteristic	n (%)
Sex	
Male	287 (64.1)
Female	161 (35.9)
Age in years, mean (SD)	64.6 (17.3)
Monitoring time in days, mean (SD)	13.1 (13.6)
Conditions of concern	
AMS	63 (14.1)
AMS, COVID-19 droplet precautions	6 (1.3)
AMS, other conditions	2 (0.4)
Brain injury or stroke	244 (54.5)
Brain injury or stroke, AMS	51 (11.4)
Brain injury or stroke, AMS, COVID-19 droplet precautions	1 (0.2)
Brain injury or stroke, COVID-19 droplet precautions	6 (1.3)
COVID-19 droplet precautions	16 (3.6)
Other conditions	59 (13.2)
Reasons for patient discontinuation	
Behaviors improved	227 (50.7)
LTACH discharge	104 (23.2)
Device reassignment to higher-need patient	45 (10)
Emergent transfer to acute care hospital	21 (4.7)
Family or patient declined the program	7 (1.6)
Patient required 1:1 sitter	5 (1.1)
COVID-19 droplet precautions were discontinued	2 (0.4)
Patient placed on palliative or hospice care	2 (0.4)
Net put in place of monitoring device	2 (0.4)
Monitoring hardware or software error^a^	33 (7.4)

AMS = altered mental status; LTACH = long-term acute care hospital.

a Includes instances when monitoring devices malfunctioned (n = 3), vendor software prompted an unexpected software update (n = 3), and vendor software experienced an unknown error (n = 20) that prompted the continuous video monitoring system to “discharge” patients being monitored.

Note: Because of rounding, some percentages may not sum to 100.

**Adverse events avoided**. One or more potential adverse events of concern were indicated for each patient at the time of enrollment. A majority (77.9%) were assigned to continuous video monitoring because, at least in part, there was a potential fall risk. More specifically, the patient distribution regarding potential adverse events of concern was falls (59.4%), falls and medical device interference or dislodgment (15.2%), and medical device interference or dislodgment (15.2%). Other such events of concern included elopement and being placed on COVID-19 precautions. For more details, see Table 2.

**Table 2.** Potential Adverse Events of Concern at Time of Program Enrollment

Potential Adverse Events of Concern	n (%)
Falls	266 (59.4)
Falls, medical device interference or dislodgment	68 (15.2)
Falls, elopement	11 (2.5)
Falls, medical device interference or dislodgment, elopement	3 (0.7)
Falls, COVID-19 precautions	1 (0.2)
Medical device interference or dislodgment	68 (15.2)
COVID-19 precautions	6 (1.3)
Elopement	5 (1.1)
Other	20 (4.5)
Total	448 (100)

Note: Because of rounding, some percentages may not sum to 100.

The telemonitor technicians recorded each time they prevented or redirected an unsafe patient behavior that had the potential to lead to an adverse event––in other words, each time their action led to an adverse event avoided. Over the 20-month study period, 7,037 adverse events avoided were recorded. Adjusted for patient time in the program, this translates to 1,198 adverse events avoided per 1,000 monitored patient-days. Falls represented the vast majority (90.7%) of adverse events avoided. The remainder of the adverse events avoided included 476 (6.8%) device dislodgments, 87 (1.2%) elopements, 22 (0.3%) instances of physical or verbal mistreatment, and 68 (1%) other such events. If a given unsafe situation could not be redressed, the technician sounded an alarm bell, alerting floor staff that they were needed in the room. Once responding staff arrived, the technician cancelled the alarm bell. The device recorded the time from alarm initiation to cancellation as the response time. The mean response time was 3.49 seconds (range, 0.10-28.77 seconds). See Table 3.

**Table 3.** Adverse Events Avoided Over the Study Period

Adverse Events Avoided	n (%)	Per 1,000 Monitored Patient-Days^a^	Response Time in Seconds, Mean (SD)
Falls	6,384 (90.7)	1,086.6	3.53 (2.27)
Medical device interference or dislodgments	476 (6.8)	81.0	3.59 (3.13)
Elopements	87 (1.2)	14.8	2.98 (1.54)
Other	68 (1)	11.6	2.71 (1.43)
Physical or verbal mistreatment	22 (0.3)	3.7	3.13 (1.77)
Total	7,037 (100)	1,197.7	3.49 (2.37)^b^

a Total 1,000 patient-days on continuous video monitoring = 5.875.

b Response times for adverse events avoided ranged from 0.10 to 28.77 seconds.

**Inpatient falls**. There were 343 total inpatient falls during the historical reference period, with a mean of 4.9 falls per 1,000 patient-days. In contrast, during the study period, inpatient falls declined significantly, with a total of 257 recorded falls, representing a mean of 3.8 falls per 1,000 patient-days. This equates to an overall 25.1% decrease in inpatient falls during the study period *(P =* 0.02). For all falls, the proportion of falls with injury during the reference and study periods were not significantly different, at 29 (8.5%) and 27 (10.5%), respectively. See Table 4.

**Table 4.** Inpatient Falls During the Reference and Study Periods

Inpatient Falls	Reference Period	Study Period	Absolute Difference	Statistic; *P* Value
Total falls, n	343	257	86	*z* = 3.51; < 0.001
Falls with injury, n (%)	29 (8.5)	27 (10.5)	2	χ^2^ (*df*) = 0.73 (1); 0.39
Falls without injury, n (%)	314 (91.6)	230 (89.5)	84	
Falls/month, mean (95% CI)	17.2 (14.5-19.8)	12.9 (10.5-15.2)	4.3 (0.86-7.7)	*t* (*df*) = 2.5 (38); 0.02
Monthly falls/monthly 1,000 patient-days, mean (95% CI)	4.9 (4.2-5.6)^a^	3.8 (3.1-4.4)^b^	1.1 (0.2-2.1)	*t* (*df*) = 2.4 (38); 0.02

a Reference period: mean (SD) monthly 1,000 patient-days = 3.482 (0.206).

b Study period: mean (SD) monthly 1,000 patient-days = 3.404 (0.131).

**Figure 1.**
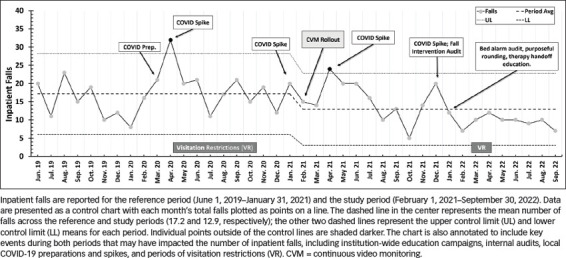
Inpatient Falls in the Reference and Study Periods

Looking at the mean fall rate by month, there were significantly fewer falls during the study period compared to the reference period, at 12.9 and 17.2 mean falls per month, respectively (*P* = 0.02). With the exception of April and December of 2021 (when there were local surges of COVID-19), each month of the study period had fewer falls than the reference period. See Figure 1.

Of the 257 falls during the study period, only 27 (10.5%) occurred in the presence of a telemonitor technician. The remaining 230 falls occurred without a technician present. Of these, 157 falls (68.3%) involved patients who were never assigned a continuous video monitoring device, as they did not meet the general requirements for a 1:1 sitter; 43 falls (18.7%) occurred among patients who were enrolled in the program only after a fall; 20 falls (8.7%) involved patients who were enrolled in the program but fell while outside their room; and 10 falls (4.3%) occurred among patients who had been enrolled in the program but whose device had been reassigned to a higher-need patient before the fall.

**Figure 2.**
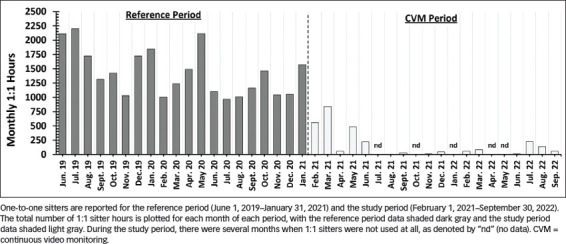
One-to-One Sitter Hours in the Reference and Study Periods

**1:1 sitter hours and cost analysis**. With implementation of the continuous video monitoring program, total 1:1 sitter hours decreased from 28,560 total hours in the reference period to 2,800 total hours in the study period, representing a 90.2% change. Looking at the monthly records, the mean number of 1:1 sitter hours decreased significantly from 1,428 hours per month in the reference period to 140 hours per month in the study period *(P <* 0.001). Similarly, there was a significant decrease in mean 1:1 sitter hours per 1,000 patient-days, from 411 hours per 1,000 patient-days during the reference period to 40 hours per 1,000 patient-days during the study period *(P <* 0.001). This translates to a decrease from a mean of 8.2 1:1 sitter FTEs during the reference period to a mean of 0.8 1:1 sitter FTEs in the study period (*P* < 0.001). See Figure 2 and Table 5.

**Table 5.** One-to-One Sitter Use

Sitter Use	Reference Period	Study Period	Absolute Difference	Statistic; *P* Value
Total 1:1 sitter hours, no.	28,560	2,800	25,760	*z* = 145.5; < 0.001
1:1 sitter hours/month, mean (95% CI)	1,428.0 (1,237.5-1,618.5)	140.0 (33.3-246.6)	1,288.0 (1,076.8-1,499.2)	*t* (*df*) = 12.4 (38); < 0.001
Monthly 1:1 hours/monthly 1,000 patient-days, mean (95% CI)	411.3 (354.2-468.5)^a^	40.3 (10.1-70.6)^b^	371.0 (308.4-434.6)	*t* (*df*) = 12.0 (38); < 0.001
Monthly FTEs, mean (95% CI)	8.2 (7.1-9.3)	0.8 (0.19-1.4)^c^	7.4 (6.2-8.6)	*t* (*df*) = 12.4 (38); < 0.001

FTE = full-time equivalent.

a Reference period: mean (SD) monthly 1,000 patient-days = 3.482 (0.206).

b Study period: mean (SD) monthly 1,000 patient-days = 3.404 (0.131).

c Does not include the 4.2 FTEs devoted to fully staffing the telemonitor technician position.

For the cost analysis, the aforementioned cost categories were considered. During the reference period, when accounting for 1:1 sitter hours only, the estimated cost was $713,988; this increased to an estimated $12,844,183 when the costs associated with inpatient falls were included. During the study period, the estimated costs associated with 1:1 sitter hours, continuous video monitoring equipment leasing, and telemonitor technician support summed to $553,754; this increased to an estimated $9,642,559 when including the costs associated with inpatient falls. In comparing the two periods, when not including costs associated with inpatient falls, the program led to estimated cost savings of $160,234, a reduction of 22.4%; when including such costs, the estimated cost savings increased to $3,201,624, a reduction of 24.9%. See Table 6.^15^

**Table 6.** Cost Analysis

Costs	Reference Period	Study Period	Absolute Difference
1:1 Sitter costs^a^	$713,988	$69,994	$643,994
Equipment leasing costs^b^	N/A	$178,000	–$178,000
Telemonitor technician costs^c^	N/A	$305,760	–$305,760
Estimated costs of inpatient falls^d^	$12,130,195	$9,088,805	$3,041,390
Estimated total costs without falls	$713,988	$553,754	$160,234
Estimated total costs with falls	$12,844,183	$9,642,559	$3,201,624

a Calculated based on a weighted average hourly rate of $25, which accounts for shift differentials and overtime pay. During the reference and study periods, 28,560 and 2,800 1:1 sitter hours were recorded, respectively.

b Calculated based on an average annual expense of $89,000 per year for two years.

c Calculated based on an average hourly rate of $21. During the 20-month study period, the telemonitor technician position was staffed full time at 4.2 FTEs, which equates to 728 hours per month and 14,560 total hours for the study period.

d Calculated using the average direct cost per inpatient fall of $35,365, as reported by Dykes and colleagues.^15^ During the reference and study periods, 343 and 257 inpatient falls were recorded, respectively.

**Patient satisfaction**. During the study period, 742 patient satisfaction surveys were returned to the study site. Of these, 29 former patients (3.9%) indicated that they'd had continuous video monitoring during their stay, and their responses were shared with the research team. Asked whether their experience with this had been positive, 15 of the 29 (52%) responded in the affirmative; of those 15, 13 (87%) had been hospitalized for brain injury or stroke. The participants reporting a positive experience indicated that they understood the need for such monitoring and were satisfied with the overall experience and the care provided. Conversely, 12 of the 29 (41%) reported that they had not had a positive experience and expressed such concerns as the lack of privacy and frustration with the technician redirecting them. Of those 12, six (50%) had been hospitalized for brain injury or stroke, four (33%) were in the general medicine patient population, and the remaining two (17%) chose not to respond to this question.

## DISCUSSION

**Main findings**. This study demonstrated that continuous video monitoring programs can significantly reduce inpatient falls in the LTACH setting and may lead to cost savings for the institution. In this study, most of the patients enrolled in the continuous video monitoring program were considered to be at high fall risk. Over the 20-month study period, more than 90% of the adverse events avoided through the use of continuous video monitoring were potential falls. While it's impossible to know whether some of the potential adverse events avoided would have occurred without this monitoring, this program was crucial in redirecting or preventing the escalation of these behaviors of concern. The significant reduction in inpatient falls during the study period, in comparison to the reference period, reflects the program's effectiveness. Moreover, most of the falls that occurred during the study period involved patients who were not under continuous video monitoring, suggesting that expanding the program could further reduce falls and other potential adverse events in the LTACH setting.

The continuous video monitoring program also significantly impacted 1:1 sitter use, with the number of 1:1 sitter hours decreasing 10-fold from the reference to the study period. This finding indicates that a continuous video monitoring device operated by a telemonitor technician from a central location can be an effective alternative to traditional 1:1 sitters. It also highlights the potential for substantial cost savings, as was demonstrated by the cost analysis.

The findings of the current study align well with those of several prior studies assessing the effect of continuous video monitoring on patient safety.^2^,^9^,^10^,^13^,^14^,^16^,^17^ For example, in a multisite study by Quigley and colleagues, the researchers found that the use of interactive “patient-engaged” video monitoring reduced the need for patient monitoring in terms of FTEs by 92%.^14^ Another study, conducted in one short-term acute care hospital, reported a 54% reduction in falls and a 72% reduction in sitter usage when both continuous video monitoring and specific nursing protocols to address patient safety were implemented.^17^ Similarly, a retrospective cohort study conducted at four short-term acute care hospitals demonstrated a 39.15% reduction in injurious inpatient falls following the introduction of a continuous video monitoring program.^16^

The current study builds upon these findings, as continuous video monitoring yielded significant reductions in inpatient falls and 1:1 sitter hours, and did so for the first time in the LTACH setting. These reductions also showed that such monitoring can optimize resource allocation and reduce costs. Together with the findings of prior studies, this study's findings demonstrate the value of continuous video monitoring as crucial to enhancing patient safety and improving economic efficiency in both short-term acute care hospital and LTACH settings.

**Telemonitor technician insights**. At the end of the 20-month study period, the four technicians currently on staff were invited to sit for an in-person interview regarding the program; two of the four accepted the invitation. Two open-ended questions were asked about what their experience as a telemonitor technician had been like and what feedback they would give to other hospitals wanting to adopt a continuous video monitoring program. Overall, the technicians were enthusiastic about the program and its positive results, and found great value in their role. They reported feeling that their primary goal was to protect the patients and to support the patients and clinical staff. Many developed personal connections with the patients they monitored. They highlighted the importance of actively listening with empathy and understanding their patients' emotional and psychological struggles, particularly during the pandemic when isolation measures were in place. It is possible that when, by doing so, technicians created a better rapport with their patients, this made the patients more likely to respond to redirection when asked.

The technicians also noted some challenges. Despite being able to communicate with patients using the continuous video monitoring system's two-way audio feature, they sometimes found it difficult to humanize these interactions. They recommended adding two-way audiovisual capability to such systems. Another challenge was that, during patient emergencies when floor staff had to be called, a technician might subsequently feel isolated, as now they had to rely solely on the floor staff for swift action. As such, it's paramount that technicians and floor staff build mutual trust and respect. Lastly, the technicians recognized that the role may not be suitable for everyone, as it requires a balance of alertness, objectivity, and the ability to multitask. This was confirmed through discussions with the nursing leadership, who stated that candidates should understand the importance of the position and be able to stay engaged and vigilant when monitoring patients, especially during the night shifts.

**Unanticipated benefits**. Although it was hypothesized that the continuous video monitoring program would lead to fewer inpatient falls, reduced 1:1 sitter hours, and result in cost savings, the study revealed several unanticipated benefits of the program as well. First, the interactive technology eased patients' social isolation during the COVID-19 pandemic, when mandated droplet precautions and visitation restrictions meant that many patients had little or no social contact for long periods of time. Second, and more surprising, there were several instances when technicians observed and alerted floor staff to visitors verbally or physically mistreating patients, events that might otherwise have gone unnoticed and unreported. In this way, the program further improved patient safety.

**Lessons learned and considerations for further research**. The following “lessons learned” may be useful to individuals and facilities wishing to implement a similar program in the future. First, a multidisciplinary approach is vital to the success of the program. To promote this, new floor staff orientation and general staff training should include a thorough explanation of continuous video monitoring and how such a program works. This will facilitate a better understanding of the telemonitor technician role and its limitations, as well as how such monitoring can complement the efforts of floor staff.

Second, at least one IT staff member should be added to the program team to assist with troubleshooting technical issues, such as Wi-Fi connectivity issues. If the monitoring devices and technician stations are in continual use, it will be necessary to plan and coordinate device and workstation restarts in order to properly install and integrate software updates. Prolonged delays in doing so can result in software glitches, which could negatively impact patient monitoring and data reporting accuracy. It is recommended that the telemonitor technicians, floor staff, IT personnel, and equipment vendor coordinate to schedule updates during periods of reduced patient activity.

Third, because this was an observational study, it wasn't feasible to collect direct patient feedback while the patient was in the monitoring program. Instead, as noted above, the research team was dependent on results from the patient satisfaction surveys that were mailed to patients after their discharge. The low response rate may have been due, at least in part, to the same diseases and conditions that made them eligible for the program. Many of the patients assigned a continuous video monitoring device had complex medical conditions that might have precluded them from completing the survey, or even recalling that they had been so monitored. In the future, it will be important to use validated measures to assess patient satisfaction with the technology, prospectively and directly.

Fourth, as this study was conducted at a single LTACH site, the findings may not be generalizable. A multisite LTACH study is recommended. That said, considering the large sample population, the extended observation period, and robust evidence of statistically significant differences between the reference and study periods, the research team is confident in recommending a similarly structured program at other health care sites treating similar populations.

Fifth, because of the impulsivity of the patient population at the study site, the appropriate ratio of telemonitor technicians to patients monitored was determined to be 1:12. This limited the number of patients who could be enrolled in the program at a time, often creating a wait list. In settings with less impulsive populations, this ratio may differ. It's recommended that each facility work with the vendor to determine the most appropriate ratio for its patients. Future studies could also investigate the impact of updated technologies, including those that incorporate new artificial intelligence tools and two-way audio–video monitoring, which might enhance communication and trust between patients and telemonitor technicians. Given the finding that the interactivity of the monitoring system helped to mitigate patients' social isolation during the pandemic, researchers should consider exploring this further.

Finally, the research team recognizes that continuous video monitoring programs may have other benefits beyond the scope of this study and recommends that other sites consider such possibilities. Future studies might explore whether such a program impacts some medically complex patient populations differently than it does others. In any case, tailoring continuous video monitoring interventions to meet the needs of specific populations will likely optimize patient care.

## CONCLUSIONS

As the findings showed, patients and the hospital both benefited from the implementation of the continuous video monitoring program. The significant reduction in inpatient falls and 1:1 sitter hours from the reference period to the study period resulted in reduced costs while maintaining high-quality patient care and improving patient safety. These results suggest that implementing a continuous video monitoring program is a cost-effective way to reduce inpatient falls, decrease 1:1 sitter use, and improve patient safety in the LTACH setting.
